# Pedestrian Stride-Length Estimation Based on LSTM and Denoising Autoencoders

**DOI:** 10.3390/s19040840

**Published:** 2019-02-18

**Authors:** Qu Wang, Langlang Ye, Haiyong Luo, Aidong Men, Fang Zhao, Yan Huang

**Affiliations:** 1 School of Information and Communication Engineering, Beijing University of Posts and Telecommunication, Beijing 100876, China; wangqu@ict.ac.cn; 2 Beijing Key Laboratory of Mobile Computing and Pervasive Device, Institute of Computing Technology Chinese Academy of Sciences, Beijing 100190, China; yelanglang@ict.ac.cn; 3 School of Software Engineering, Beijing University of Posts and Telecommunication, Beijing 100876, China; zfsse@bupt.edu.cn; 4 State Key Laboratory of Advanced Optical Communication Systems and Networks, Peking University, Beijing 100871, China; huangyan0910@pku.edu.cn

**Keywords:** indoor positioning, deep learning, pedestrian dead reckoning, walking distance, stride-length estimation

## Abstract

Accurate stride-length estimation is a fundamental component in numerous applications, such as pedestrian dead reckoning, gait analysis, and human activity recognition. The existing stride-length estimation algorithms work relatively well in cases of walking a straight line at normal speed, but their error overgrows in complex scenes. Inaccurate walking-distance estimation leads to huge accumulative positioning errors of pedestrian dead reckoning. This paper proposes TapeLine, an adaptive stride-length estimation algorithm that automatically estimates a pedestrian’s stride-length and walking-distance using the low-cost inertial-sensor embedded in a smartphone. TapeLine consists of a Long Short-Term Memory module and Denoising Autoencoders that aim to sanitize the noise in raw inertial-sensor data. In addition to accelerometer and gyroscope readings during stride interval, extracted higher-level features based on excellent early studies were also fed to proposed network model for stride-length estimation. To train the model and evaluate its performance, we designed a platform to collect inertial-sensor measurements from a smartphone as training data, pedestrian step events, actual stride-length, and cumulative walking-distance from a foot-mounted inertial navigation system module as training labels at the same time. We conducted elaborate experiments to verify the performance of the proposed algorithm and compared it with the state-of-the-art SLE algorithms. The experimental results demonstrated that the proposed algorithm outperformed the existing methods and achieves good estimation accuracy, with a stride-length error rate of 4.63% and a walking-distance error rate of 1.43% using inertial-sensor embedded in smartphone without depending on any additional infrastructure or pre-collected database when a pedestrian is walking in both indoor and outdoor complex environments (stairs, spiral stairs, escalators and elevators) with natural motion patterns (fast walking, normal walking, slow walking, running, jumping).

## 1. Introduction

Accurate and pervasive indoor positioning significantly improves our daily life [1]. The demand for accurate and practical location-based services anywhere using portable devices, such as smartphones, is quickly increasing in various applications, including asset and personnel tracking, health monitoring, precision advertising, and location-specific push notifications. A recent market report predicted that the global indoor-location market size is expected to grow from $7.11 billion in 2017 to $40.99 billion by 2022, at a Compound Annual Growth Rate of 42.0% during the forecast period [2].

To meet this explosive demand, various indoor positioning approaches have recently been developed, including RFID [3], Wi-Fi [4,5], UWB [6], BLE [7], magnetic [1,8,9,10], visible light [11,12] and visual methods [13]. Indoor localization techniques are classified into propagation model-based, fingerprint, and dead reckoning methods. Positioning performance of propagation model-based methods depends on the deployment density of the reference points. However, these methods are ineffective when the radio signal is weak or not available in many scenarios, such as underground parking lots. The accuracy of fingerprint-based approaches is affected by device orientation, pedestrians, and layout changes of indoor environments (e.g., removing or adding furniture). It is important that fingerprint- or infrastructure-based positioning techniques are not available for emergency scenarios, such as anti-terrorism action, emergency rescues and exploration missions.

Inertial Measurement Unit (IMU, consisting of gyroscopes and accelerometers)-based navigation algorithms overcome the limitations of radio-frequency signal fluctuations and blockage since they provide real-time locations of a pedestrian or object given an initial position, as well as not relying on any additional infrastructure or pre-collected database. To date, the previous studies on IMU-based navigation algorithms fall into two typical mechanizations: strap-down inertial navigation systems (INS) and pedestrian dead reckoning (PDR) [14].

INS mechanization provides more high-frequency and richer navigation information, including pedestrians’ 3D position, velocity, and attitude by integrating raw data from accelerometers and gyroscopes. However, positioning errors rapidly increase with time due to the drift characteristics of IMU sensors. To constrain the accumulative distance error of double integration, a zero-velocity-aided inertial navigation system uses periodic zero-velocity updates [15] by mounting an IMU module to the foot (such as toes, instep, or heel) of pedestrians. To restrain the accumulation errors of heading, zero angular rate updates [16] and magnetic angular rate updates [17] have been used to eliminate gyroscope bias. To date, the best foot-mounted INS (FM-INS) controls walking-distance estimation error in 0.3% of the entire travel distance [18]. However, FM-INS needs an additional foot-mounted inertial module as the platform, as well as special protection solutions (e.g., shockproofing, waterproofing, and pressure prevention) are necessary to protect the module from damages. Therefore, it is challenging to implement an FM-INS for extensive use in the consumer market [14].

**Figure 1:**
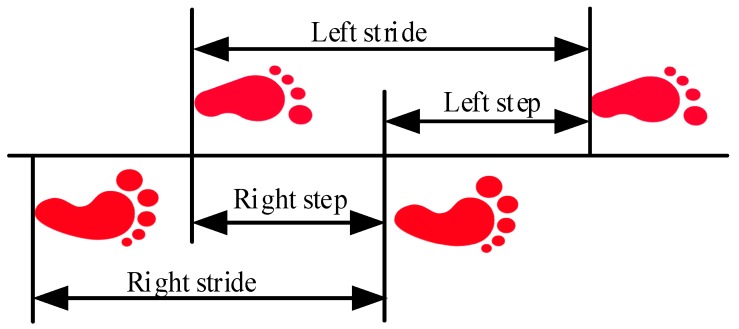
Strides are defined by the positions of two consecutive footfalls of the same foot, while the steps are defined by the positions of opposite feet.

The smartphone-based PDR mainly benefits from the extensive use of smartphones—most consumers always carry smartphones that have integrated inertial-sensor. PDR without the restriction of only using foot-mounted sensors estimates the relative location of a pedestrian by joining heading estimation, step detection, and SLE (stride-length estimation). As shown in Figure 1, strides are defined by the positions of two consecutive footfalls of the same foot, while the steps are defined by the positions of opposite feet. More details of the gait description can be found in [19]. The AHRS [20,21] provides robust and high-precision heading estimation for PDR by fusing the information from IMU sensors and magnetometers. An ocean of studies about step detection and step count have been done, including peak detection [22], zero-crossing [23], auto-correlation [24], and neural networks [25]. All of them achieve superior step-count accuracy. Stride-length is simultaneously computed using the generalized formulas related to the magnitudes of the accelerometers and gyroscopes, step frequency, and height of the pedestrian. SLE has a significant impact on the performance of PDR systems. Moreover, a pedestrian’s stride-length provides beneficial information for human gait analysis, sports-activity monitoring, prediction the status of human health, or energy-consumption assessment [26]. Nowadays, to develop PDR systems with smartphone embedded low-cost sensors, accurate SLE over a long period is a crucial problem for the following reasons [27]: (1) Stride-length differs with height, gender, age and weight; (2) Even the same person’s stride-length significantly varies depending on environmental differences and walking patterns (fast walking, normal walking, slow walking, running and jumping); (3) It is difficult to keep the stride-length constant even for the same person walking in a single pattern, in a single environment. The methods for estimating pedestrian step length are summarized as two categories: one is direct methods based on the integration of the acceleration; the other is indirect methods that leverage a model or assumption to compute step length.

The double integration of the acceleration component of the sagittal axis, is, in theory, the best method to compute the stride-length of pedestrians because it does not rely on any model or assumption, and it does not require training phases or individual information (leg length, height, weight) [28]. Kourogi [29] leveraged the correlation between walking velocity and vertical acceleration to estimate walking speed, then calculated step length by multiplying walking speed by step interval. Kang et al. [30] simultaneously measured the inertial sensor and global positioning system (GPS) position while walking outdoors with a reliable GPS fix, and regarded the velocity from the GPS as labels to train a hybrid multiscale convolutional and recurrent neural network model. After that, Kang leveraged the prediction velocity and moving time to estimate the traveled distance. However, it is challenging to obtain accurate labels since GPS contains a positional error. Moreover, it is difficult to obtain the acceleration component in the forward direction from the sensor’s measurements, as well as constantly maintain the sensor heading parallel to the pedestrian’s walking direction [31].

Considerable studies based on model or assumption have been conducted to improve the accuracy of stride-length estimation (SLE), and are summarized as empirical relationships [32,33], linear models [34], nonlinear models [35,36,37,38], biomechanical models [35,38], context-based [39,40] regression-based [41,42,43,44] and neural network [45,46]. A general pointer [47] had systematic compared of different methods for estimating short linear displacements of the body. Weinberg [36] leveraged the maximum and minimum acceleration values on the Z-axis in each stride to estimate walk distance. Kim [37] developed an empirical method that utilized the average of the acceleration amplitude in each step during walking to calculate the movement distance. To accurately estimate the traveled distance of a pedestrian, Ladetto [34] leveraged the linear relationship between step length and frequency, and the local variance of acceleration amplitude, to calculate motion distance. The methods mentioned above are only based on the maximum and minimum of acceleration amplitude, without considering the orientation and position of the smartphone. Gao [39] and Martinelli [40] proposed context-based step length estimation method However, the limited context classification cannot reflect the diversity of pedestrian walking patterns and environments. Zihajehzadeh and Park [43,44] leveraged Lasso regression to fit the linear model by minimizing a penalized version of the least squares loss function. However, it is difficult for handcraft-extracted features or models to reflect the walking patterns of a pedestrian. To solve this problem, Cho [46] utilized the neural-network method for step length estimation. Hannink [45] described a novel approach to stride-length estimation with deep convolutional neural networks and achieved better accuracy. However, this method relied on the stride-specific inertial-sensor that were attached to the pedestrian’s feet. Therefore, this method is unsuitable for smartphone-based applications.

These SLE algorithms present good performance in the case of walking a straight line at normal speed. Most of them however suffer from various limitations, such as unsuitability for smartphones, limited attitude, and relying on individual information or spatial constraints. However, a pedestrian may walk arbitrarily in different directions and may stop from time to time. Moreover, real paths including turns, stairs, sidesteps, varying speed or actions performed by the subject result in unacceptable SLE accuracy.

Deep learning has the ability to automatically learn features with high-level abstractions by using multiple nonlinear transformations [48]. Deep learning has been proven to be powerful for natural language processing and speech-recognition, without additional processing. Convolutional neural networks and Recurrent Neural Networks show excellent potential to exploit and analyze the collected data without handcraft feature extraction. Motivated by the fact that speech recognition based on deep learning outperforms other existing traditional speech recognition methods, this paper proposed a stride-length estimation method based on Long Short-Term Memory (LSTM) and Denoising Autoencoders (DAE), termed TapeLine, to address the challenge of PDR systematic errors caused by motion distance estimation from a natural walk in complex conditions. TapeLine estimated pedestrian’s stride-length and walking-distance with inertial-sensor embedded in smartphones. The key contributions of our study are as follows:

- We propose a training frame for combining LSTM and DAE to deal with sequential data to extract the temporal feature meanwhile denoise, and a stride-length estimation model based on the training frame. Since the inertial-sensor measurements are time series data, we leveraged LSTM to excavate the temporal dependencies and extract significant features vectors from noisy inertial-sensor measurements. Denoising Autoencoders were adopted to automatically sanitize the inherent noise and obtain denoised feature vectors. A regression module was employed to map the denoised feature vectors to the resulting stride length.
- We trained the proposed model with walking information from a smartphone, and the ground truth of stride-length from a foot-mounted IMU module, to predict an adaptive stride-length. In addition to the raw inertial-sensor data, the high-level stride-length features based on the excellent early studies are directly fed to the merge layer of networks. The proposed method is free of the zero-velocity assumption that double-integration methods need to reinitialize the integration process and eliminate accumulative errors.
- In addition to evaluate the robustness performance of the proposed TapeLine under different operation conditions, we compared TapeLine with the existing commonly-used stride-length estimation methods in both single-stride and complex paths. Whether stride-length estimation or walking-distance estimation in complex environments with natural walking patterns, our proposed method outperformed commonly-used stride-length estimation methods and achieved a superior performance, with a stride-length error rate of 4.63% and a walking-distance error rate of 1.43%.
- We established a benchmark dataset with ground truth for training step counting and stride-length estimation. A foot-mounted IMU module (x-IMU [49] controls motion distance errors in 0.3% of the entire travel distance) was attached to a pedestrian’s shoes that provided precise heel strike times and actual stride-length. Training data was generated from smartphone, and the annotated data were generated by a foot-mounted IMU module. In addition to model training and performance evaluation of step counting and stride-length estimation, the dataset is applied to explore the optimal parameters.

The rest of the paper is organized as follows: in Section 2, we detail the solution of the proposed TapeLine. In Section 3, we describe the benchmark dataset collection in detail and evaluate the proposed scheme. In Section 4, we provide a conclusion that summarizes the importance of our proposed work.

## 2. Materials and Methods

In this section, an overview of the system is depicted. Then, the key modules and important algorithms are described, which include data pre-processing based on LSTM-DAE and adaptive stride-length estimation.

### 2.1. System Architecture

**Figure 2:**
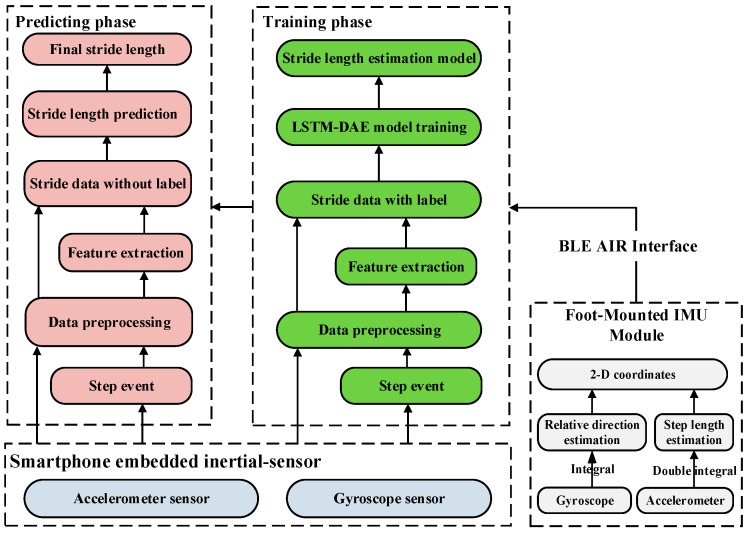
System architecture of the proposed TapeLine.

Figure 2 illustrates the overall structure of TapeLine. A smartphone is used to collect the inertial data that is split into segments, with each segment representing the data for one stride. We utilized the inertial-sensor measurements from the smartphone as training data, and the corresponding motion distances from the FM-INS module (x-IMU [49] with a three-axis accelerometer (range ±16 g, resolution 490 μg), and a three-axis gyroscope (range ±2000 deg/sec, resolution 0.06 deg/sec) from x-io technologies) as labels to train a predictive model in the offline phase. In the online predicting phase, we leveraged the real-time sensor data and trained model to adaptively estimate the length of each stride.

### 2.2. Benchmark Dataset

**Figure 3:**
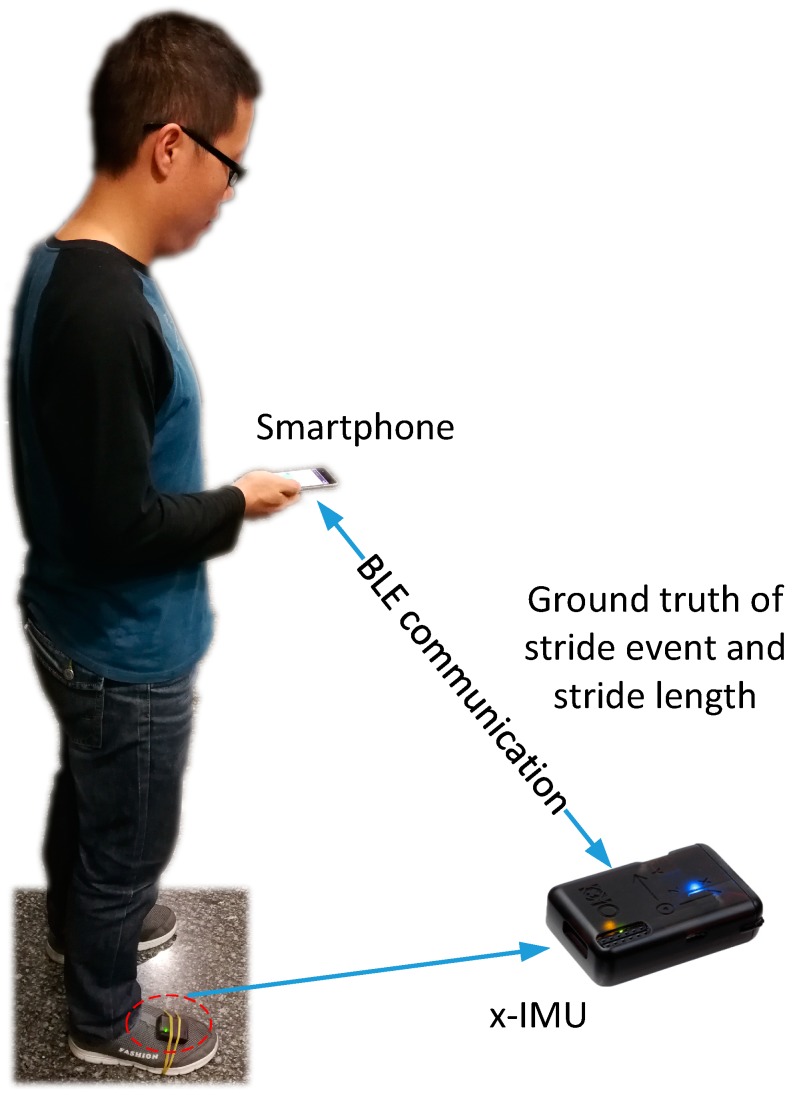
Training data collection system.

The lack of benchmarking datasets for pedestrian stride-length and walking-distance makes it hard to pinpoint the differences of the published methods. The existing datasets either lack the ground-truth of each stride or are limited to small spaces with a single-scene or motion pattern. It is natural to think of using laser rangefinder to measure the motion distance of each stride. However, it is laborious or impossible to measure the stride-length of numerous strides using a laser rangefinder. To thoroughly evaluate the performance of stride-length and walking-distance estimation, we used our benchmark dataset (https://github.com/Archeries/StrideLengthEstimation/ tree/master/Benchmark-Dataset-for-Adaptive-Stride-Length-Estimation) for natural pedestrian dead reckoning using a smartphone (Huawei Mate 9 with an 8-core 2.4 GHz processor) and an FM-INS module (x-IMU [49] with a three-axis accelerometer (range ±16 g), and a three-axis gyroscope (range ±2000 deg/sec) from x-io technologies), as shown in Figure 3. The smartphone inertial-sensors (a three-axis accelerometer (range ±8 g), and a three-axis gyroscope (ICM-20690, range ±2000 deg/sec) from InvenSense (Sunnyvale, CA, USA) were sampled at 100 Hz. The FM-INS module performs zero-velocity updates algorithm to detect heel strike moment of foot attached FM-INS module, and then calculates motion distance of the foot by acceleration integration, and sends motion distance to smartphone via Bluetooth. The x-IMU module only sends motion distance to mobile phone, which guarantee the real-time performance of Bluetooth communication. x-IMU controls walking-distance estimation error in 0.3% of the entire travel distance, while the mean of stride (two steps) length is 1.36 m. The single stride error of x-IMU is less than 0.4 cm (1.36 m *0.3% = 0.00408 m ≈ 0.4 cm). Therefore, the FM-INS module is precise enough to serve as ground truth. Once a stride is detected, the sampling application on smartphone will automatically associate the collected sensor data with motion distance. The datasets were gathered by five volunteers (three males and two females, aged between 23 and 32, height between 152 and 196 cm, and weight between 45 and 80 kg) with natural motion patterns (including, fast walking, normal walking, slow walking, running, jumping). Throughout the datasets, the users hold the phone in their right hand in front of their chest. The datasets contain more than 14 km and 10,000 strides of gait measurements. The datasets contain indoor and outdoor cases, including stairs, escalators, elevators, office environments, shopping malls, streets, and metro stations. To maximize the compatibility, all data were published in open and simple file formats. The sample hold nine degree-of-freedom sensor data from smartphone embedded sensors and the corresponding stride count, stride-length, and cumulative walking-distance from foot-mounted module attached to the instep of the right foot of the pedestrian. To make it easier for readers to replicate experiment, we shared the sampling software in GitHub (https://github.com/Archeries/StrideLengthEstimation/tree/master/SampleAPP) More detailed info about the dataset and the sample app can be found in the readme.md file (https://github.com/ Archeries/StrideLengthEstimation) of GitHub.

### 2.3. Data Preprocessing and High-Level Feature Extraction

**Figure 4:**
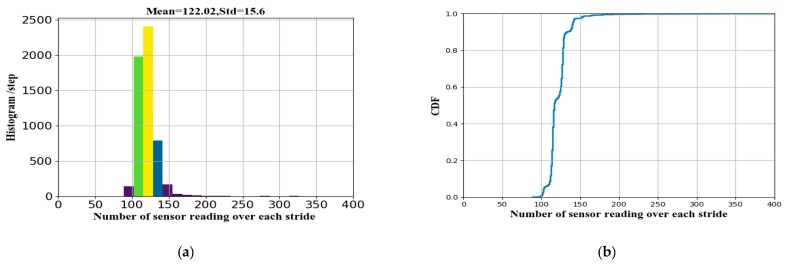
Distribution of sensor-reading number over each stride. (**a**) histogram; (**b**) cumulative distribution.

Before data were fed to the LSTM network, we performed a series of preprocessing operations, including data segmentation, extraction of annotated strides, and length alignment. First, we split the inertial sensor data according to the step event. For each segment, we extracted the sensor data and corresponding ground truth of the stride-length to generate the training data and labels. We normalized the readings from the accelerometer and gyroscope with respect to the respective sensor ranges. Figure 4a shows the histograms of the sensor-reading number over each stride. From the figure, we can see that the mean and deviation of the stride-length are 122.2 and 15.6, respectively. We inferred that the average time for each stride was 1.22 s since the sampling rate was set to 100 Hz. Figure 4b demonstrates that the sensor-reading number of the 99th percentile of each stride was less than 200. Therefore, 300 samples (3 s) of each stride were enough to cover different walking speeds except static. To ensure equally scaled and fixed-size input to the network, we infinity-padded or intercepted the sensor samples per stride to a fixed length of 300. Preprocessed data *x_i,j_* contain *i* = 1, …, 300 samples and *j* = 1, …, 6 channels.

After data preprocessing, we extracted higher-level features based on excellent early studies including Weinberg [36], Kim [37], Ladetto [34] and Scarlett [50] according to the following equations:

(1) $$Weinberg=K\times \sqrt[{4}]{a_{\max}-a_{\min}}$$

(2) $$Kim=K\times \sqrt[{3}]{\frac{\sum_{i=1}^{N}\left|a_{i}\right|}{N}}$$

(3) Ladetto=α×f+β×v+γ

(4) $$Scarlett=K\times \frac{\frac{\sum_{i=1}^{N}\left|a_{i}\right|}{N}-a_{\min}}{a_{\max}-a_{\min}}$$

where *K* denotes the calibration coefficient that is obtained by the ratio of the real distance and the estimated distance. $a_{\max}$ and $a_{\min}$ denote the maximum and minimum acceleration values on the Z-axis in each stride, respectively. $a_{i}$ represents the measured acceleration value of the $i^{th}$ sample in each stride. *f* and *v* denote step frequency and acceleration variance, respectively. *α* and *β* denote the weighting factors of step frequency and acceleration variance, respectively. *γ* represents a constant.

**Figure 5:**
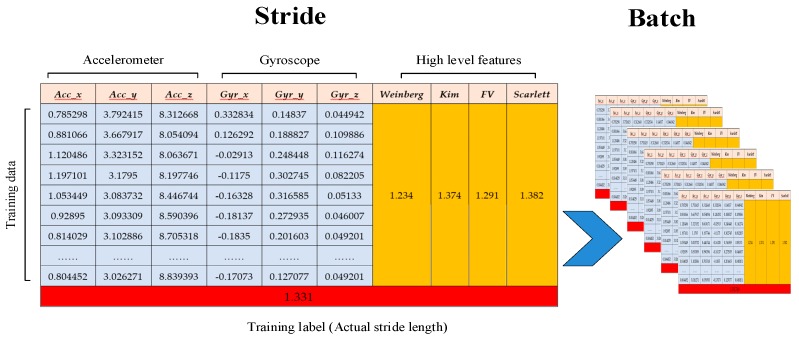
Input example for a stride-length estimation network.

As shown in Figure 5, we construct stride data using the six channels of raw inertial information (the silver numbers) over a stride as well as the four extracted high-level features (the yellow numbers). The red number is the motion distance of each stride from the FM-INS module. After that, the constructed stride data will be fed to the neural network described later.

### 2.4. Stride-Length Estimation Model

**Figure 6:**
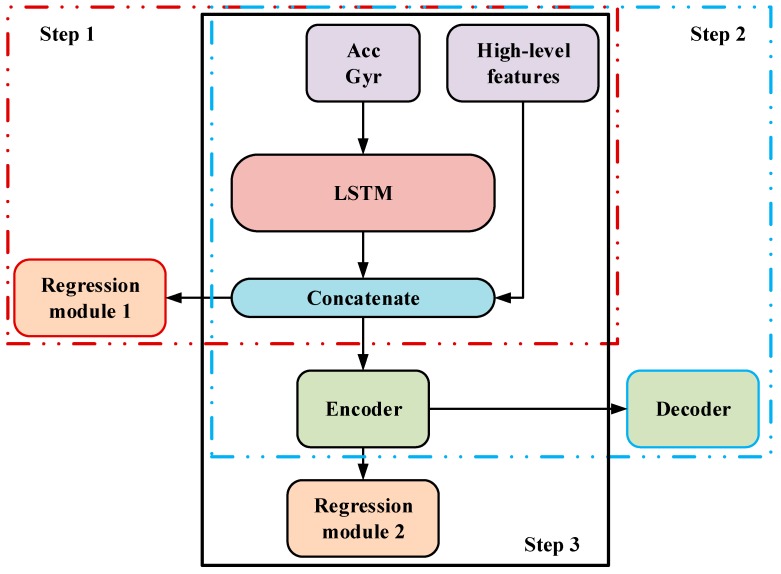
The proposed training frame.

Our proposed solution for stride-length estimation is composed of three parts for three steps, as shown in Figure 6: (1) A pure LSTM model which contains two LSTM layers and four fully connected layers, which is first trained to get a relatively optimal weights distribution of LSTM. (2) A DAE model including the two LSTM layers in (1), keeping them untrainable, and a Dropout-Encoder-Decoder module, which is trained to get a noise-robust encoder. (3) The final regression model built by adding a regression module with three fully connected layers to the encoder we got, of which all the layers are fine-tuned.

#### 2.4.1. Temporal Feature Extraction based on Long Short-Term Memory

The measurements of sensors (i.e., accelerometer, gyroscope) over a stride (two consecutive steps) are expressed as follows:

(5) $$a_{i}={\left[\begin{array}{ccc} ax & ay & az \end{array}\right]}^{T}$$

(6) $$g_{i}={\left[\begin{array}{ccc} gx & gy & gz \end{array}\right]}^{T}$$

(7) $$Acc_{i}=\left[\begin{array}{cccc} a_{1} & a_{2} & \cdots & a_{T} \end{array}\right]$$

(8) $$Gyr_{i}=\left[\begin{array}{cccc} g_{1} & g_{2} & \cdots & g_{T} \end{array}\right]$$

(9) $$D=\left\{\left(\left[Acc_{i},Gyr_{i}\right],y_{i}\right),i=1,\cdots ,N\right\}$$

where the segment size T is fixed constant. *a_x_*, *a_y_* and *a_z_* indicates the tri-axis accelerometer. *g_x_*, *g_y_* and *g_z_* indicates the tri-axis gyroscope.

In a LSTM cell, **x***_t_*, **h***_t_* and **C***_t_* represent the input vector, the recurrent hidden state, and the long-term state which keeps the long-term memory of cell by gates (forget, input and output), at time-step *t*, respectively. LSTM takes the acceleration measurements in stride as the input sequences, and feeds to the cell sequentially. The outputs of gates at time-step *t* are first calculated as Equations (10)–(12). For *t* = 0, both the previous cell state and hidden layer are assigned to zero vectors:

(10) $$f_{t}=\sigma \left(W_{f}\cdot \left[h_{t-1},x_{t}\right]+b_{f}\right)$$

(11) $$i_{t}=\sigma \left(W_{i}\cdot \left[h_{t-1},x_{t}\right]+b_{i}\right)$$

(12) $$o_{t}=\sigma \left(W_{o}\cdot \left[h_{t-1},x_{t}\right]+b_{o}\right)$$

where **W** is M×(M+3) weight matrix. **b** is a *M* × 1 bias matrix.σ(·) is a sigmoid function providing nonlinearity for gates.

Then the candidate state value ${\tilde{C}}_{t}$ is calculated as (13), and the long-term state $C_{t}$ is updated as (14):

(13) $${\tilde{C}}_{t}=\tanh\left(W_{C}\cdot \left[h_{t-1},x_{t}\right]+b_{C}\right)$$

(14) $$C_{t}=f_{t}\circ C_{t-1}+i_{t}\circ {\tilde{C}}_{t}$$

The output of current time-step is calculated as (16), and so as the final output, which should be an embedding of the input sequence in temporal feature space:

(15) $$h_{t}=o_{t}\circ \tanh\left(C_{t}\right)$$

(16) $$H\left(x\right)=h_{T}=o_{T}\circ \tanh\left(C_{T}\right)$$

**Figure 7:**
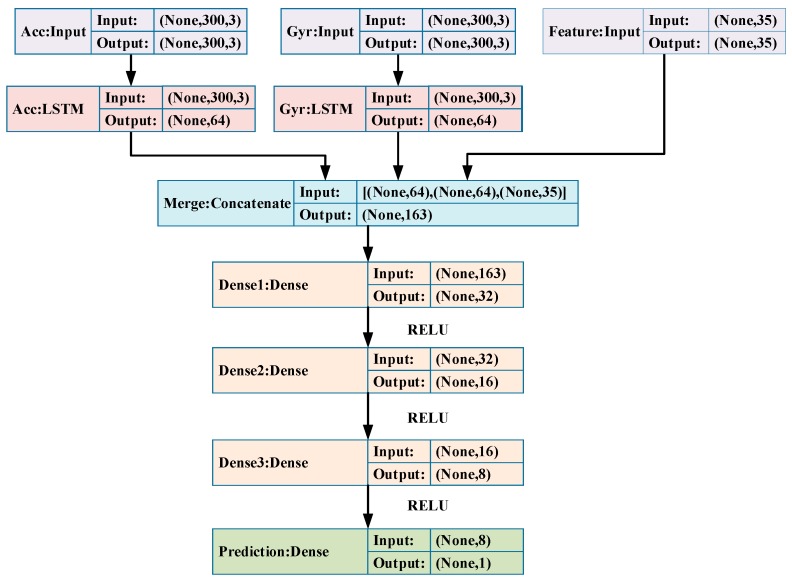
The architecture and parameters of the pure LSTM model.

The architecture and parameters of layers of the pure LSTM model are shown in Figure 7. As we can see, the LSTM model takes accelerometer and gyroscope measurements as well as the high-level features mentioned above. The hidden Dense layers have Rectified Linear Unit (ReLU) as their activation function, and the prediction Dense layer has no activation function cause it’s a regression task. The LSTM layers map the input sequences to an embedding in temporal feature space, while the high-level features provide expert knowledge for the model. A concatenate layer is added to merge these information. Then, four cascading fully connected layers are added to build a map between feature space and target value (i.e., the stride-length).

#### 2.4.2. Noise sanitization based on Denoising Autoencoders

Due to the fact the raw sensor readings from accelerometers and gyroscopes inevitably contain noise, it is necessary to perform a filtering operation. Unlike the traditional filtering techniques (e.g., low-pass or median filter), the denoising autoencoders approach is highly efficient in learning signal features and predicting stride-length with much better accuracy. In order to force the hidden layer to extract more robust features and prevent it from simply learning the identity, we trained a DAE (Denoising Autoencoders) to reconstruct the merged feature from a corrupted version of it.

**Figure 8:**
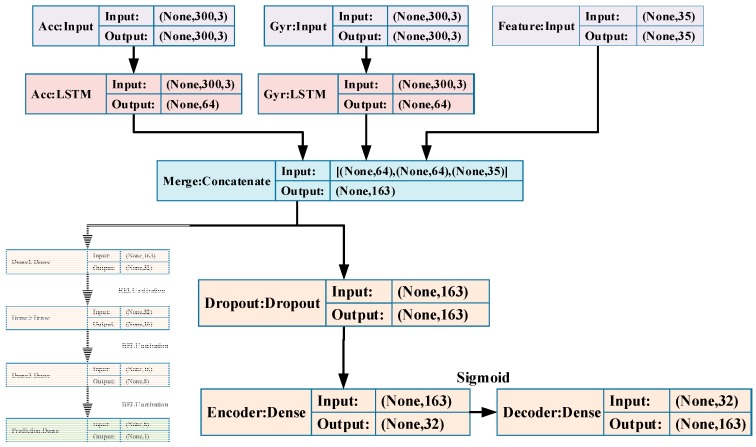
The architecture and parameters of DAE model.

As shown in Figure 8, the DAE contains three parts: a Dropout, an Encoder and a Decoder. The network output maps the hidden representation of corrupted **h** back to a reconstruction $\hat{h}$. Our DAE model takes the merged feature **h** as the input and corrupts it by a Dropout layer Drop(·), then the Encoder Enc(·) maps it to a lower dimension space and Decoder Dec(·) maps it back to merged feature space. After that, the hidden features learned from autoencoders are utilized as inputs for the regression model for stride-length estimation:

(17) $$h=\left[\begin{array}{c} H\left(Acc\right)\\ H\left(Gyr\right)\\ Feats \end{array}\right]$$

(18) $$\hat{h}=\mathrm{Dec}\left(\mathrm{Enc}\left(\mathrm{Drop}\left(h\right)\right)\right)$$

Given a merged feature h, the DAE trains the Encoder and Decoder to minimize the reconstruction error, which corresponds to minimize the following objective function:

(19) $$J_{DAE}\left(h,\hat{h}\right)=\frac{1}{2M}\sum_{i=1}^{M}{\left(h_{i}-{\hat{h}}_{i}\right)}^{2}$$

#### 2.4.3. Stride-Length Regression

**Figure 9:**
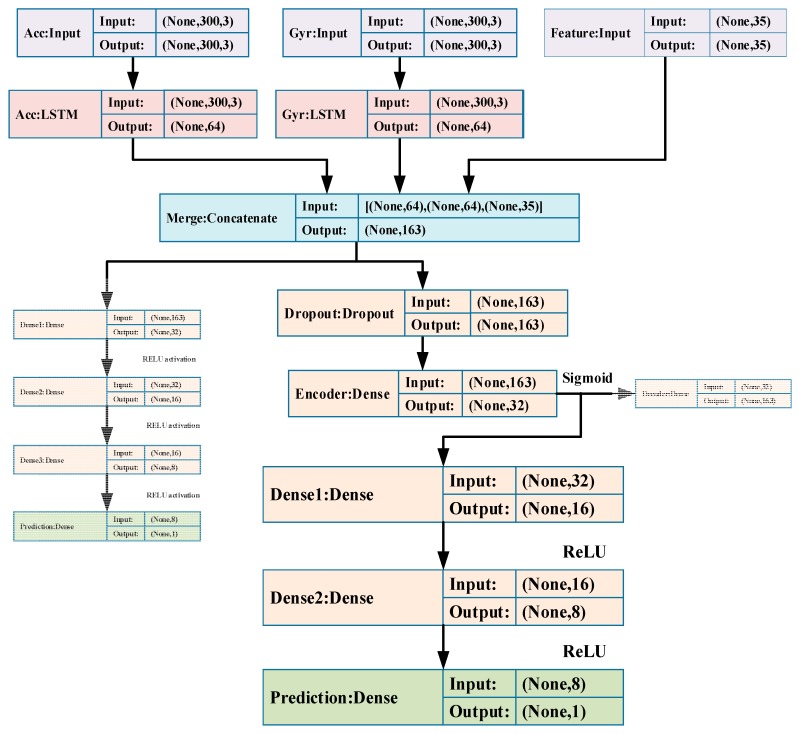
The architecture and parameters of final regression model.

As shown in Figure 9, once the DAE is built, the actual stride-length is utilized to train the supervised regression layer that estimates the stride-length of pedestrian. The global objective of the regression layer is to minimize the error loss function *J*(**D**,G) between the actual stride-length and the estimated value:

(20) $$\hat{y}=G\left(\mathrm{Enc}\left(\mathrm{Drop}\left(h\right)\right)\right)$$

(21) $$J\left(D,G\right)=\frac{1}{2N}\sum_{i=1}^{N}{\left(y_{i}-{\hat{y}}_{i}\right)}^{2}$$

where *y_i_* is the actual stride-length corresponding to the input D. ${\hat{y}}_{i}$ is the stride-length estimation from the regression layer. G(·) is the regression module in the final regression model. The minimization is realized by the RMS propagation which often used in the training of neural networks.

Algorithm 1 depicts the complete procedures of the proposed stride-length estimation method. The algorithm takes a set of training samples with the corresponding actual stride-length as input to train the network. The accelerometer readings and gyroscope readings are divided into segments by stride event. The fixed length of stride data is fed to LSTM-DAE network. The actual stride-length is used to train the regression layer on the top of the network. Once the training is done, the LSTM-DAE model will be used to predict the stride-length of pedestrian.


**Algorithm 1. adaptive stride-length estimation based on LSTM-DAE**
1**Input:** training data with actual stride-length $T=\left\{t_{i},i=1,2,\cdots ,n\right\}$, test data without actual stride-length2**Output:** stride-length estimation of pedestrian 3
***// Data preprocessing***
4Split the inertial sensor data according to the stride event.5**For** each stride **do**6   Extract sensor data and corresponding ground truth to generate the training data and labels7   Extract high-level feature8   Infinity-pad or intercept the sensor samples of per stride to a fixed length9   Construct Stride data as shown in Figure 510
**End for**
11
***// Model training***
12build and train the pure LSTM model13build the DAE model and initialize the weights of LSTM layers by the pure LSTM model, set the LSTM layers to be untrainable and train DAE model14build the final regression model and initialize the weights of layers before Decoder, set all layers to be trainable and train to fine-tune15
***//Testing***
16Leverage trained model to predict stride-length of pedestrian

### 2.5. Parameter Set and Network Performance

Our proposed TapeLine algorithm was implemented using Keras (https://keras.io/) with pandas for data management in a Windows environment. Optimization was performed with the RMS propagation (RMSprop) algorithm [51]. Table 1 summarizes the hyperparameter values of TapeLine.

**Figure 10:**
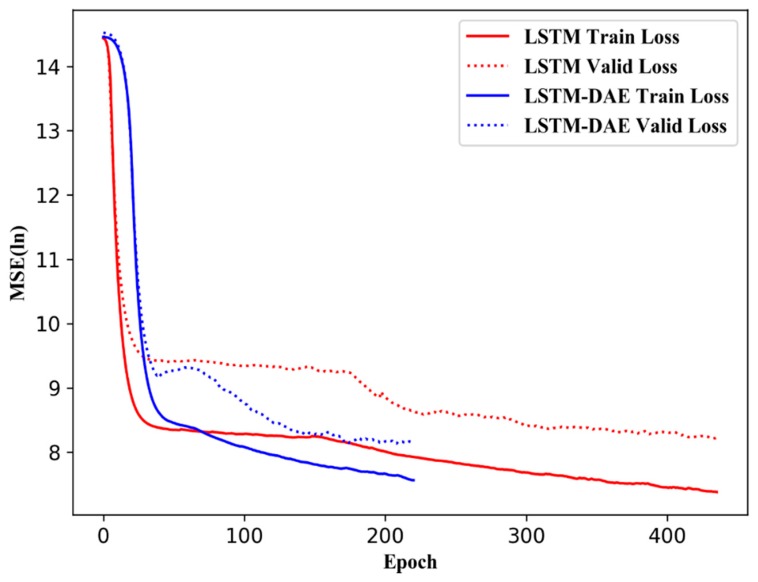
Cost function with respect to training and the test data set.

To prevent over-fitting, we employed a dynamic stop criterion for the model training. System automatically stop the iteration when the loss function does not drop within 50 epochs. Figure 10 illustrates the variation in loss function with respect to training and validation. As the figure shows, the loss function for training data tends to decrease with the iterations, whereas that for validation decreases to a certain iteration and then slightly increases again. Stride length estimation based on pure LSTM needed 436 epochs to reach a stable regime on the entire training dataset, whereas stride length estimation based on LSTM-DAE only need 221 epochs. Stride length estimation based on LSTM-DAE converges faster than that based on pure LSTM.

### 2.6. Walking-Distance Estimation

To improve the robustness of the step counting, we utilized the step counting algorithm proposed by Kourosh [52], which solves the overcounting problem caused by false walking (e.g., when users use their phones for playing games in a still state). The cumulative walking-distance of pedestrians is calculated with the number of steps and estimated stride-length in every valid step event. Denoting N as the cumulative number of strides walked during the experiment, we established walking-distance *CD* by summing the adaptive stride-length of each stride as follows:

(22) $$CD=\sum_{i=1}^{N}L_{i}$$

where *L_i_* represents the length of the *i*-th stride.

### 2.7. Evaluation Metrics

We leveraged the stride-length error rate and walking-distance error rate to measure the proposed method. The relative error rate of the stride-length is calculated with the following formula:

(23) $$E_{s}=\frac{1}{N}\sum_{i=1}^{N}\left(\frac{\left|L_{e}^{i}-L_{t}^{i}\right|}{L_{t}^{i}}\times 100\%\right)$$

where $L_{e}^{i}$, $L_{t}^{i}$ denote the estimated stride-length and the actual stride-length of the *i*-th stride, respectively.

The relative error rate of walking-distance is calculated with the following formula:

(24) $$E_{cd}=\frac{\left|\sum_{i=1}^{M}L_{e}^{i}-\sum_{i=1}^{M}L_{t}^{i}\right|}{\sum_{i=1}^{M}L_{t}^{i}}\times 100\%$$

where $L_{e}^{i}$, $L_{t}^{i}$ denote the estimated stride-length and the actual stride-length of the *i*-th stride, respectively.

## 3. Experimentation and Evaluation

To understand the effectiveness and limitations of the proposed TapeLine algorithm, we implemented and evaluated TapeLine in both indoor and outdoor complex environments (stairs, spiral stairs, escalators and elevators) with natural motion patterns (fast walking, normal walking, slow walking, running, jumping).

### 3.1. Experimental Setup

We collected test data using an Android smartphone (Huawei Mate 9 with an 8-core 2.4 GHz processor), which equipped with a three-axis accelerometer (range ±8 g), and a three-axis gyroscope (range ±2000 deg/sec) from InvenSense (ICM-20690). Both single stride-length error and accumulative stride-length error are essential indicator that used to evaluate the accuracy of stride-length algorithms. In Section 3.2, we first utilize the single stride-length error to compare the proposed TapeLine with the state-of-the-art SLE methods and verify the robustness of the proposed TapeLine among different scenarios, heterogeneous devices and different pedestrians using the control variable method, respectively. In Section 3.3, we utilized accumulative stride-length error to evaluate the accuracy of TapeLine in an indoor-outdoor complicated path and an indoor closure path.

### 3.2. Experiment Results of Stride-Length Estimation

We leveraged the trained SLE model and learnable parameters (e.g., weights and biases) to predict stride-length. In addition to comparing the proposed stride-length estimation with the state-of-the-art SLE methods, we also conducted extensive experiments to verify the robustness of the proposed TapeLine in different scenarios, with heterogeneous devices, and different pedestrians using the variable-controlling approach, respectively.

#### 3.2.1. Comparison of Stride-Length Estimation using LSTM and LSTM-DAE

To explore how much performance improvement was gained from DAE, we trained two SLE models (LSTM-based, and LSTM and DAE-based) using the same training dataset and test data. Table 2 summarizes stride-length estimation comparison of LSTM and LSTM–DAE. 80% of the stride-length error and error rate were 0.063 m and 4.63%, respectively. Compared to LSTM, mean error rate reduced from 3.75% to 3.16%. In another word, the mean error rate of LSTM-DAE was reduced by 15.7% ((3.75%–3.16%)/3.75%). The experiment results demonstrated that DAE help improved stride-length estimation accuracy.

#### 3.2.2. Comparison with Other Methods

**Figure 11:**
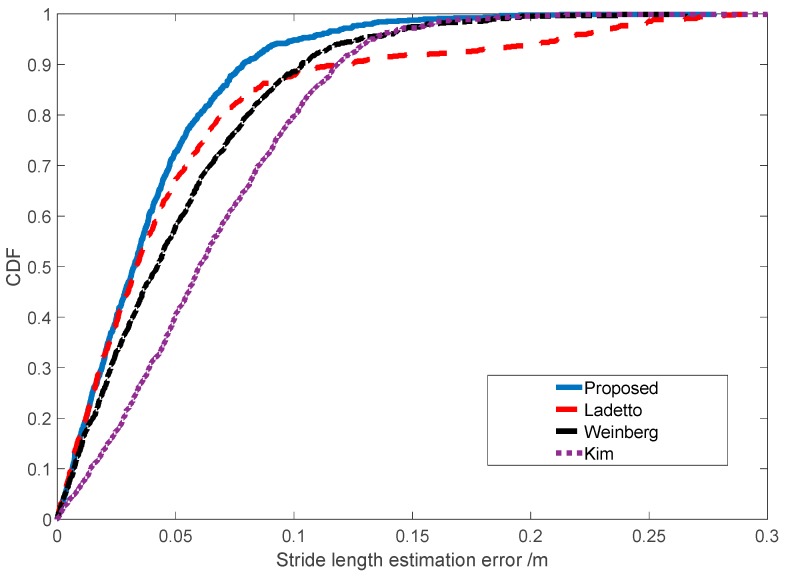
The estimation error of proposed model and other methods.

**Figure 12:**
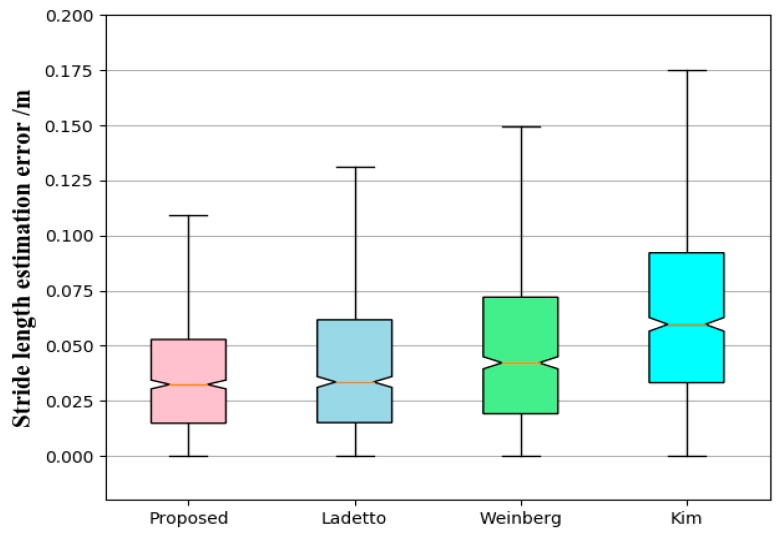
Box plot of stride-length estimation error. In this box plot, the whiskers represent the 99.3% coverage. On each box, the central (red) mark is the median, the edges of the box are the 25th and 75th percentiles.

We also compared the proposed stride-length estimation method with the state-of-the-art SLE methods (Kim [37], Ladetto [34] and Weinberg [36]). To clearly illustrate the distribution of SLE errors, we used box plots and CDF to compare the statistics of single SLE errors (total 1000 strides with natural walking). From Figure 11, we can see that the relative error of the proposed algorithm is smaller than those achieved by the Kim, Weinberg, Ladetto algorithms. From Figure 12, we can see that the median, the lower and the upper quartiles of the proposed algorithm are smaller than those of the Kim, Weinberg, Ladetto algorithms.

#### 3.2.3. Robustness among Typical Scenarios

**Figure 13:**
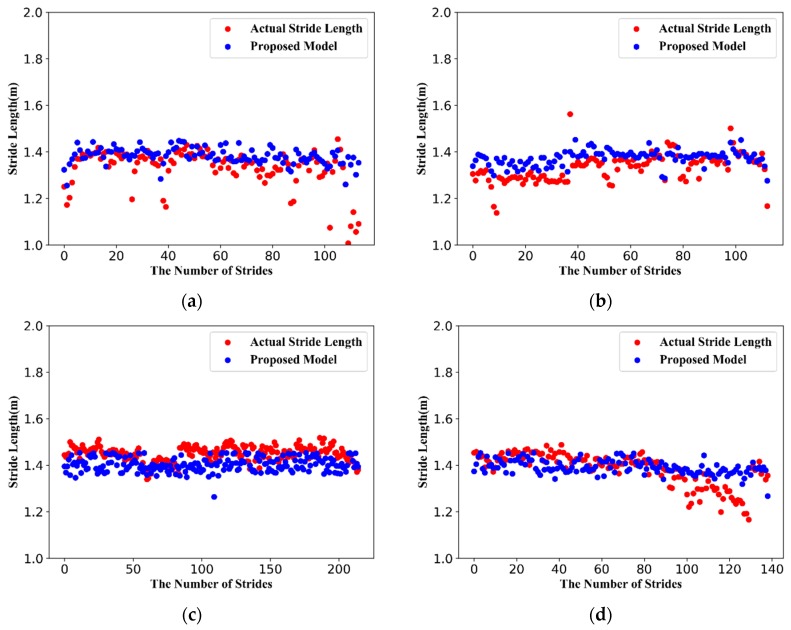

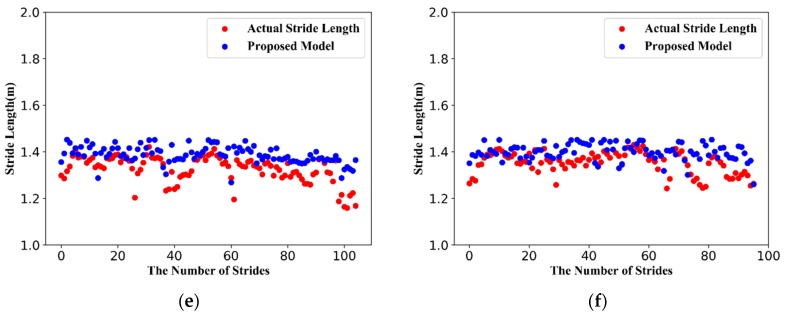
Comparison of estimated stride-length and true stride-length in typical scenarios. (**a**) offices; (**b**) shopping malls; (**c**) metro stations; (**d**) underground parking lots; (**e**) streets; (**f**) footpath.

To verify the performance and practicality of our proposed method, we conducted experiments with six different settings that covering a wide range of typical scenarios (offices, shopping malls, streets, metro stations, underground parking lots, streets, and footpaths). Figure 13 shows the comparison of estimated stride-length and actual stride-length.

**Figure 14:**
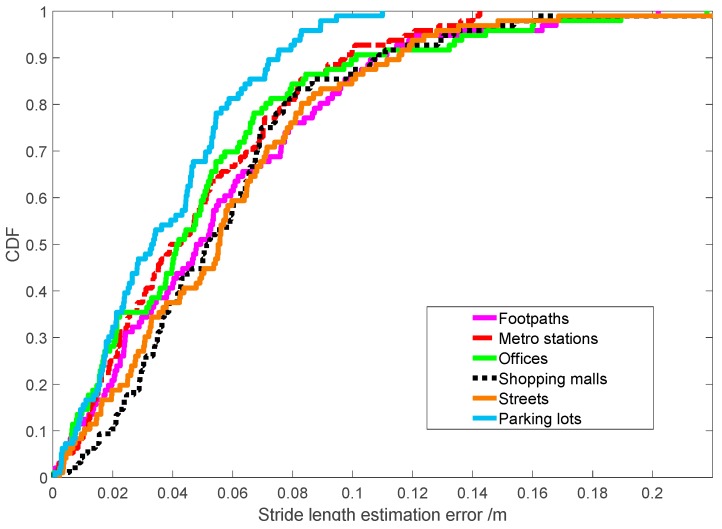
Estimation error of stride-length in typical scenarios.

The CDF (cumulative distribution function) of the SLE error for all scenarios is plotted in Figure 14. All results demonstrate that it achieves promising performance, where 80% of the stride-length estimation errors occur within 0.071, 0.076, 0.075, 0.058, 0.082 and 0.088 m, and 80% of the error rates occur within 5.22%, 5.59%, 5.51%, 4.26%, 6.03%, and 6.47%, respectively.

#### 3.2.4. Robustness among Heterogeneous Devices

**Figure 15:**
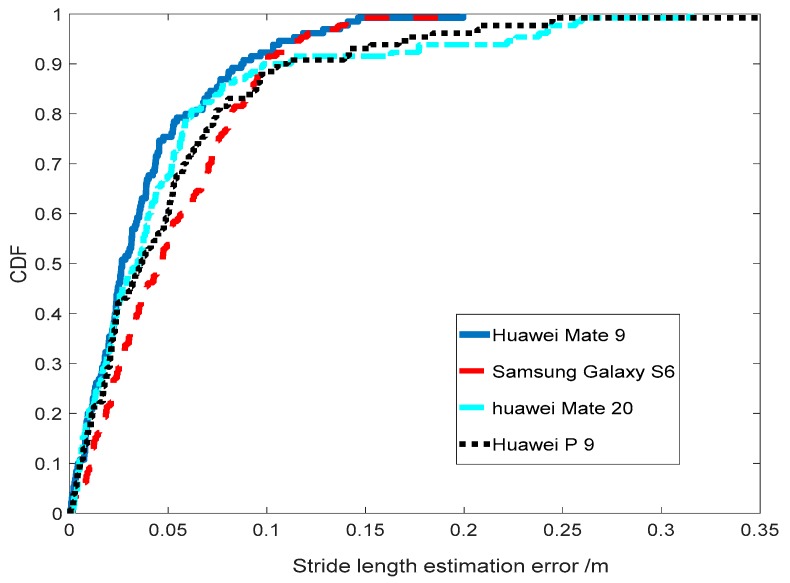
Robustness among different devises.

Device heterogeneity is a long-standing common challenge of indoor localization techniques. Even under identical environment, different devices would observe different inertial-sensor signals due to the hardware diversity and inevitable sensors noise. To examine TapeLine’s robustness, we conducted experiments using a data acquisition device (Huawei Mate 9) and another three heterogeneous devices (Samsung Galaxy S6, Huawei Mate 20, and Huawei P 9). The CDF of stride-length estimation error for four devices is plotted in Figure 15. From the figure, we can see that the CDF curves are very close, and they are consistent with the experimental results of data acquisition device. This demonstrates the robustness and practicality of TapeLine.

#### 3.2.5. Robustness among Different Pedestrians

**Figure 16:**
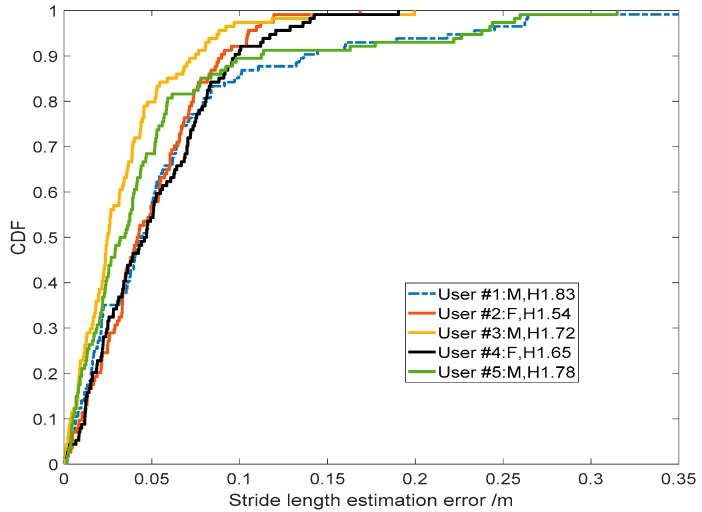
Robustness among different pedestrians.

The above experiment results were performed by two of our authors. To evaluate the robustness of TapeLine with respect to different pedestrians that have different walk patterns, we invited five other volunteers. Naturally, their gait patterns were different. To accurately record the actual stride-length of pedestrians, an FM-INS module was attached to the volunteers’ instep of the right foot. All the volunteers were assigned to walk along the same path (about 400 m) in the office environment. The CDF of the SLE error for all five volunteers is plotted in Figure 16. From the figure, we can see that the five CDF curves are very similar, and they are consistent with the experiment results from our own walks. The experiment results demonstrate the robustness and practicality of TapeLine.

### 3.3. Walking-Distance Estimation in Complex Paths

**Figure 17:**
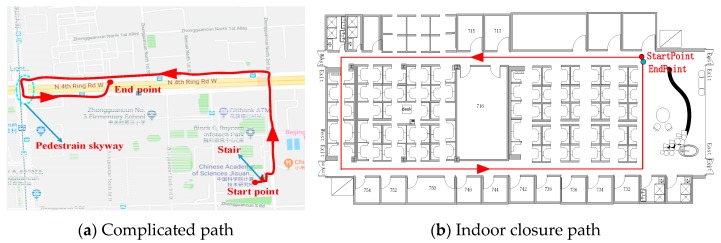
Walking path description. The volunteers were asked to travel the highlighted path with a natural pattern. Volunteer 1 (172 cm, 66 kg) walked along path (**a**). Volunteer 2 (180 cm, 80 kg) walked along path (**b**).

To evaluate the accuracy of TapeLine under various conditions with natural walking patterns, we started walking from an indoor office (the seventh floor of the Institute of Computing Technology, Chinese Academy of Sciences). After walking for about 100 meters, we reached the stairs. We walked downstairs from the seventh floor to the ground floor. Then, we exited the office and walked along streets to the youth apartment of the Chinese Academy of Sciences. The path length was about 1265 meters and 907 strides, including a pedestrian skyway. Figure 17a illustrates the entire walking path. We also invited a new volunteer using a new smartphone to conduct similar experiment along an indoor closure path (see Figure 17b). The comparison of walking-distance estimation is shown in Table 3.

**Figure 18:**
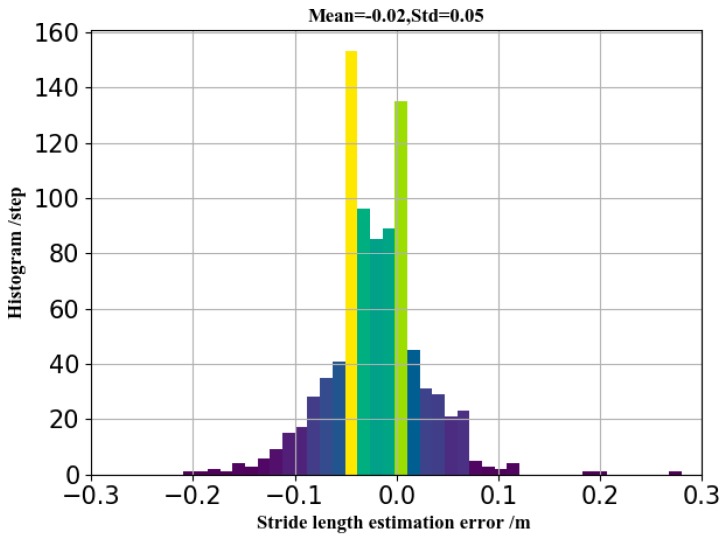
Estimation error distribution of stride-length.

Whether the proposed or compared methods, the walking-distance error rate is significantly less than the stride-length error rate. To explore the reason, we visualized the error distribution of stride-length estimation as Figure 18.

From the figure, we can see that the stride estimation error follows a Gaussian distribution. Therefore, the cumulative stride-length error is less than the sum of the single stride-length error.

### 3.4. Time Complexity Analysis

The most time-consuming procedures of TapeLine are the training data collection and neural-network training. However, both of these two procedures are performed in the offline phase, which means they do not consume any time during the online prediction phase. The proposed method was implemented in Python and performed on a personal computer equipped with an Intel Core i5-4460 CPU at 3.20 GHz and 16 GB of DDR4 RAM. Table 4 reports the training and test time of TapeLine with different network models on the whole training dataset and test data. The time consumption of the training data collection equals walking time. For a 14 km and 10,000 strides data set, the time consumption of training is 3 h 01 min 26 s. From our test, the running time of prediction was less than 2.7 ms (2.369 s/888) for each stride.

## 4. Discussion and Conclusions

Pedestrian stride-length provides beneficial information for human gait analysis, sports-activity monitoring, and the prediction status of human health. Especially, SLE is one of the most critical factors in PDR-based indoor positioning. Accurate SLE of pedestrians is a challenging research topic, due to the various walking patterns. Since LSTM examined the temporal dependencies and extracted significant features vectors from noisy inertial-sensor measurements, and Denoising Autoencoders automatically sanitized the inherent noise and obtained denoised feature vectors, the proposed model adapts to the characteristics of different pedestrians and their walking patterns. In addition to single stride-length estimation, we also conducted walking-distance estimation experiments in challenging scenarios, including indoor and outdoor environments with natural walking patterns. The proposed method achieved a superior performance, with a single stride-length error rate of 4.63% and a walking-distance error rate of 1.43%. Generally, whether for single stride-length or walking-distance estimation, our proposed method outperforms commonly-used stride-length estimation methods. The performance of the Weinberg model and the Kim model were similar and worse than our proposed stride estimation algorithm. Both of them take the acceleration and the K parameter as input, and do not consider walking speeds or phone attitudes. By considering pedestrian’s acceleration and step frequency, the Ladetto model is more robust against different walking speeds and phone attitudes than the Weinberg model and the Kim model.

Detecting heel strike moment and splitting the inertial-sensors data are the key point for accurate stride-length estimation. In this paper, the training data is split by a foot-mounted IMU module. The training data segmentation is affected by the time delay of acceleration integration and Bluetooth communication. In practical applications, it is extremely difficult to strictly obtain sensor data at every stride by only using the smartphone built-in inertial sensor. In the offline phase, we estimate the offset between the peak point of acceleration and the heel strike moment detected by foot-mounted IMU module. In the online phase, we utilize the offset and peak point of acceleration to split the sensor data of each stride. However, the online sensor data segmentation for each stride is not accurate enough, thus resulting in inaccurate stride-length estimation. The problem of inertial-sensor data splitting and segmentation is still an open issue in all stride-length estimation methods. In our future work, we will explore more accurate tool (e.g., a high speed camera) to obtain more accurate ground truth, segment and align the sensor data of each stride.

Since humans are flexible structures, it is difficult to ensure that the movement of mobile phones equals the movement of pedestrians. Extra actions (standing still or swing hands, playing games, calling, reading, etc.) results in inaccurate stride-length estimation. Different smartphone carrying methods will have an obvious influence on stride-length estimation accuracy. Therefore, we reiterate here that pedestrians should hold their phone horizontally with the hand in front of their chest. The proposed approach was tested on a few healthy adults and devices. However, the trained model may be not suitable for children, elderly and non-healthy adults (e.g., Parkinson’s patients). In the future, we will investigate how to automatically obtain training data by crowdsourcing, then train a personalized SLE model in the form of online learning. The personalized model will undoubtedly further enhance the performance of the proposed method.

## Figures and Tables

**Table 1:** List of hyperparameter values for network models.

Parameter	LSTM	DAE	Final Model
**Batch size**	128	128	128
**Hidden layers**	32-16-8-1	32-163	32-16-8-1
**Activation**	ReLU	Sigmoid/Linear	ReLU
**Optimizer**	RMSprop [51]	RMSprop	RMSprop
**Learning rate**	0.001	0.001	0.001
**Epochs**	500	50	500
**Early stopping**	50	/	50
**Loss function**	MSE	MSE	MSE

**Table 2:** Comparison of stride-length estimation using LSTM and LSTM–DAE.

Attributes	LSTM		LSTM-DAE	
	Error	Error Rate ^1^	Error	Error Rate
**Mean**	0.051	3.75%	0.043	3.16%
**Std**	0.037	-	0.036	-
**25%**	0.025	1.83%	0.017	1.25%
**50%**	0.045	3.31%	0.036	2.64%
**75%**	0.068	5.00%	0.059	4.34%
**min**	4.38 × 10^−4^	0	5.67 × 10^−5^	0
**max**	0.340	25.00%	0.239	17.57%

^1^ According to Equation (23).

**Table 3:** Comparison of walking-distance estimation in complex paths.

Path	Attributes	Real	Proposed	Ladetto	Weinberg	Kim
**a**	**Total distance (m)**	1267.82	1249.67	1238.20	1223.40	1219.74
	**Error (m)**	-	18.15	29.62	45.42	48.08
	**Error rate ^2^**	-	1.43%	2.34%	3.50%	3.80%
**b**	**Total distance (m)**	94.43	93.01	91.75	97.57	97.69
	**Error (m)**	-	1.42	2.68	3.14	3.26
	**Error rate**	-	1.50%	2.83%	3.32%	3.45%

^2^ According to Equation (24).

**Table 4:** Comparison of time complexity.

Models	Training Dataset Size	Test Dataset Size	Trainable Parameters	Training Time	Test Time
**LSTM**	6571 strides	888 strides	40737	2 h 11 min 34 s	2.158 s
**LSTM-DAE**			92101 (40737 + 10627 + 40737)	3 h 01 min 26 s	2.369 s
